# Major Walter Reed

**Published:** 1902-12

**Authors:** 


### OBITUARY.

Major Walter Reed, surgeon U. S. Army, died at Washington, November 23, 1902, aged 51 years. His death was due to appendicitis, for which an operation was performed November 17. Major Reed was sent to Havana to investigate the yellow-fever question and it was largely through his researches that the determination was reached that the disease was communicable through the mosquito.

Dr. Reed was born in Gloucester County, Va., in 1851, and was a graduate of the medical department of the University of Virginia and of Bellevue Hospital Medical College, New York. He was appointed an assistant surgeon in the army in 1875 and at the time of his death was first on the list of surgeons with the rank of major. He had been known for years as one of the foremost bacteriologists and pathologists of the country. In 1893 he was appointed curator of the Army Medical Museum in Washington.

During the Spanish-American War he was a member of the board to investigate typhoid fever in the army. After the war he made several voyages to Cuba and was on duty in Havana, studying tropical diseases in general and yellow fever in particular. After a series of experiments, which cost the life of one member of the board, early in 1901, it was announced as a proved fact that yellow fever is conveyed by a certain variety of mosquito and introduced into the blood of non-immunes by its bite. Sanitary measures tending to the destruction of the insect and the screening of infected persons were put into effect immediately in Havana by order of General Wood, with the result that for over a year no case of yellow fever has there developed, though the disease had existed permanently in Havana for three centuries. It is impossible to estimate the value of the scientific work of this able, courageous, and self-sacrificing army surgeon. His death constitutes a national loss.

Major Reed is survived by a widow and daughter residing at Washington, and a son, Lieutenant W. L. Reed, Tenth Infantry, now in the Philippines.

